# Supplementary material for: Sweetening with Agavins: Its Impact on Sensory Acceptability, Physicochemical Properties, Phenolic Composition and Nutraceutical Potential of Oak Iced Tea
Source: Foods. 2025 Feb 28;14(5):833. doi: 10.3390/foods14050833 (PMC11898482; doi:10.3390/foods14050833)
Supplement: Supplementary file 1 [file foods-14-00833-s001.zip › foods-3487113-supplementary.pdf]

Table S1. Chromatographic and spectrometric conditions for the identification of phenolic profiling

No.	Compounds	Code	RT (min)	λmax	Nominal mass	Ions*
1.	Quinic acid	QA	0.57	274.86	191.20	93.06, ***85.06***
2.	Shikimic acid	ShA	0.63	---	173.18	***111.07***, 93.06
3.	Gallic acid hexoside iso I	GA-Hex (I)	0.71	262.86	331.06	***169.01***
4.	Galloylquinic acid iso I	GQA (I)	0.75	269.86	343.06	***191.05***, 169.05
5.	Galloylquinic acid iso II	GQA (II)	1.04	269.86	343.06	***191.05***, 169.05
6.	Gallic acid	GA	1.11	270.86	169.15	***125.05***, 79.07
7.	Dihydroxybenzoic acid hexoside	DiHBA-Hex	1.53	276.86	315.07	***152.04***
8.	Gallic acid hexoside iso II	GA-Hex (II)	1.65	263.86	331.06	***169.01***
9.	Protocatechuic acid	PA	2.15	293.86	153.15	***109.05***, 91.04
10.	Caffeoylquinic acid	CQA	2.62	325.86	353.10	***191.20***, 85.10
11.	2,5-dihydroxybenzoic acid	2,5-DiHBA	2.90	271.86, 322.86	153.15	***108.92***, 81.09
12.	Coumaric tartaric acid	CouTA	3.08	308.86	295.04	***163.02***, 118.92
13.	Hydroxybenzoic acid hexoside	HBA-hex	3.24	276.86	299.07	***136.98***
14.	4-hydroxybenzoic acid	4-HBA	3.26	276.86	137.04	***93.05***, 65.09
15.	Caffeic acid hexoside	CA-hex	3.41	324.86	341.08	***179.05***, 135.08
16.	Chlorogenic acid	ChA	3.46	325.86	353.10	***191.20***, 85.10
17.	Cryptogenic acid	CrA	3.69	325.86	353.10	***191.20***, 85.10
18.	Caffeic acid	CA	3.88	321.86	179.19	***135.08***, 89.09
19.	Sinapoylquinic acid	SQA	4.29	280.86	397.11	223.01, ***191.07***
20.	*p*-Coumaric acid	CouA	4.99	308.86	163.24	***119.08***
21.	Ferulic acid	FA	5.63	321.86	193.24	178.07, ***134.04***
22.	2-hydroxybenzoic acid	2HBA	6.36	299.86	137.04	***93.05***, 65.09
23.	Trans-cinnamic acid	t-CiA	8.48	276.86	147.17	***103.08***, 77.07
24.	Procyanidin B1	PB1	3.21	277.86	577.44	407.21, ***289.18***
25.	Catechin	C	3.60	278.86	288.97	***245.06***, 203.00, 122.96
26.	(*epi*)-catechin	*epi*-C	4.42	276.86	288.97	***245.06***, 203.00, 122.96

27.	Myricetin hexoside	M-Hex	5.17	---	479.08	***316.99***, 179.02
28.	Rutin	R	5.87	354.86	609.04	***270.94***, 254.89
29.	Quercetin 3-O-β-glucuronide	Q-glur	5.99	348.86	476.92	***300.99***, 150.96
30.	Quercetin 3-O-glucoside	Q-glu	6.10	352.86	463.36	***300.42***, 271.11, 151.00
31.	Kaempferol 3-O-glucoside	K-glu	6.66	352.86	447.30	***284.24***, 255.12
32.	Quercetin rhamnoside	QR	6.69	365.86	447.09	***301.03***, 178.99, 151.00
33.	Phloridzin dihydrate	Ph	7.39	283.86	470.96	435.05, ***272.98***
34.	Kaempferol rutinoside	KR	8.27	---	593.15	***285.03***
35.	Quercetin	Q	8.39	361.86	300.95	178.91, ***150.96***
36.	Trigalloyl glucoside	TG-glu	3.66	283.86, 382.86,	635.00	***465.00***, 313.00
37.	Ellagic acid xyloside	EA-Xyl	5.28	360.86	433.00	***301.00***, 257.00
38.	Ellagic acid rhamnoside iso I	EA-Rham (I)	5.49	360.86	447.00	***300.00***, 257.00
39.	Ellagic acid	EA	5.69	360.86	300.97	283.92, ***228.92***, 144.93
40.	Ellagic acid glucoside	EA-glu	6.03	360.86	463.00	***300.00***, 113.00
41.	Ellagic acid derivative*(k)	EA-der	6.62	360.86	447.00	***299.00***, 113.00
42.	Ellagic acid rhamnoside iso II	EA-Rham (II)	6.67	360.86, 365.86	447.00	***300.00***, 257.00
43.	Ellagic acid methyl ether	EAME	7.12	---	315.00	***300.00***, 228.92

RT denote retention time; *compounds identified on the basis of their major transitions and quantified on the base of qualifier ion highlighted in bold

Table S2. Pearson correlation analysis between the phenolic compounds, the physicochemical parameters and their response intensity by FT-IR in different key regions for formulations of *Q. sideroxyla* and different concentrations of agavins.

**Compound**	**pH**	**TTA**	**900-1200**	**1100-1250**	**1100-1330**	**1580-1610**	**3200-3450**
QA	0.954	-0.725	0.935	0.878	0.874	0.843	0.903
GQA (II)	0.931	-0.634	0.840	0.773	0.767	0.752	0.813
GA-Hex (II)	0.860	-0.776	0.863	0.844	0.852	0.828	0.816
CouTA	0.810	-0.722	0.832	0.859	0.845	0.847	0.782
GQA (I)	0.931	-0.634	0.840	0.773	0.767	0.752	0.813
2-HB	0.876	-0.641	0.807	0.720	0.750	0.708	0.765
Q-glur	-0.379	0.897	-0.536	-0.778	-0.816	-0.843	-0.388
2,5-DiHBA	0.731	-0.776	0.878	0.874	0.884	0.859	0.823
CA-Hex	0.771	-0.584	0.762	0.754	0.735	0.721	0.737
TG-Glu	-0.731	0.663	-0.897	-0.854	-0.822	-0.783	-0.875
C	-0.637	0.915	-0.751	-0.941	-0.941	-0.957	-0.631
FA	-0.715	0.732	-0.719	-0.771	-0.800	-0.788	-0.639
ShA	0.655	-0.861	0.715	0.863	0.874	0.895	0.597
CrA	-0.599	0.914	-0.689	-0.886	-0.901	-0.925	-0.564
ChA	-0.578	0.917	-0.706	-0.890	-0.906	-0.931	-0.581
Di-HBA-Hex	0.769	-0.370	0.804	0.648	0.601 p=0.03	0.546	0.844
t-CiA	0.820	-0.476	0.662	0.594	0.584	0.576 p= 0.04	0.657
CouA	-0.777	0.456	-0.639	-0.481	-0.530	-0.487	-0.589

**Compound**	**pH**	**TTA**	**900-1200**	**1100-1250**	**1100-1330**	**1580-1610**	**3200-3450**
EAME	0.736	-0.314	0.651	0.496	0.492	0.459	0.648 *p*= 0.02
CA	-0.515	0.673	-0.604 *p*= 0.03	-0.739	-0.731	-0.751	-0.490

See Table S1 for codes of phenolic compounds

Table S3. Pearson correlation analysis between the phenolic compounds, the physicochemical parameters and their response intensity by FT-IR in different key regions for formulations of *Q. eduardii* and different concentrations of agavins.

**Compound**	**pH**	**TTA**	**900-1200**	**1100-1250**	**1100-1330**	**1580-1610**	**3200-3450**
QA	0.054	-0.777	0.715	0.971	0.891	0.875	0.703
2-HB	0.165	-0.705	0.590 *p*= 0.04	0.936	0.818	0.924	0.571
ChA	-0.207	0.644	-0.517	-0.870	-0.791	-0.873	-0.493
4-HB	0.252	-0.901	0.537	0.812	0.926	0.733	0.535
CryA	-0.259	0.750	-0.485	-0.780	-0.812	-0.771	-0.492
FA	-0.086	-0.877	0.779	0.825	0.915	0.601 *p*= 0.03	0.784
ShA	0.312	-0.290	0.283	0.747	0.497	0.933	0.263
PA	-0.241	-0.754	0.793	0.793	0.786	0.559	0.780
Di-HBA-Hex	0.052	-0.730	0.554	0.744	0.750	0.625	0.553
CA-Hex	-0.021	0.729	-0.618 *p*=0.03	-0.758	-0.782	-0.610 *p*=0.03	-0.613 *p*=0.03
Q-glur	-0.348	0.626	-0.371	-0.708	-0.691	-0.724	-0.390
CouA	0.110	0.223	-0.504	-0.695	-0.406	-0.743	-0.479
R	0.403	-0.343	0.132	0.565	0.448	0.777	0.110
EA-Rham (II)	0.014	0.445	-0.422	-0.617	-0.482	-0.627	-0.406
EA-Xyl	0.034	0.307	-0.484	-0.707	-0.452	-0.700	-0.454
CQA	-0.250	0.289	-0.260	-0.655	-0.460	-0.761	-0.246
TG-Glu	-0.048	0.492	-0.409	-0.608	-0.508	-0.627	-0.403

Compound	pH	TTA	900-1200	1100-1250	1100-1330	1580-1610	3200-3450
GQA I	-0.496	-0.563	0.883	0.761	0.642	0.486	0.877
PB1	0.182	0.118	-0.574	-0.741	-0.347	-0.724	-0.558
GA-Hex I	-0.154	-0.654	0.647	0.680	0.681	0.485	0.641

See Table S1 for codes of phenolic compounds
